# What does it mean to call a medical device invasive?

**DOI:** 10.1007/s11019-023-10147-x

**Published:** 2023-05-03

**Authors:** Eran Klein

**Affiliations:** 1 Department of Neurology, Oregon Health & Science University, 3181 SW Sam Jackson Park Road, L226, Portland, OR 97239-3098 United States of America; 2 Department of Philosophy, University of Washington, Washington, United States of America

**Keywords:** Medical device, Invasiveness, Normative, Bioethics, Technology

## Abstract

Medical devices are often referred to as being invasive or non-invasive. Though invasiveness is relevant, and central, to how devices are understood and regarded in medicine and bioethics, a consensus concept or definition of invasiveness is lacking. To begin to address this problem, this essay explores four possible descriptive meanings of invasiveness: how devices are introduced to the body, where they are located in the body, whether they are foreign to the body, and how they change the body. An argument is offered that invasiveness is not purely descriptive, but implicates normative concepts of danger, intrusion, and disruption. In light of this, a proposal is offered for how to understand use of the concept of invasiveness in discussions of medical devices.

## Introduction

The concept of *medical invasiveness*, as some have called it (Rudnick 2011), is a feature of biomedical, regulatory, and bioethics discourses about medical devices.^1^ Medical devices, like pacemakers, intrauterine devices (IUDs), electrocardiograms (EKGs), and many others are commonly referred to as invasive or non-invasive (or minimally invasive or minutely invasive) (Leuthardt et al. 2021). The concept of invasiveness is applied widely to a broad range of diagnostic, therapeutic, and preventative medical devices targeting diverse organ systems (e.g., respiratory, cardiac, and neurological) and is found in discussions about how devices are developed and regulated (e.g., type of study design, level of human subject protection, threshold for regulatory approval (Ashton et al. 2009)), made accessible or distributed (e.g., by professional authorization vs. over the counter (Wexler 2016)), marketed (e.g., the competitive advantage of non- or minimally invasive technology (e.g., MySugarWatch Limited)), and used (e.g., preference for non-invasive measures in hospice care (Shao et al. 2017)). Yet despite its presence throughout discourses involving medical devices, the concept of invasiveness itself is seldom defined or clearly characterized.^2^

Consider the range of medical devices and interventions to which the concept invasive is commonly applied.^3^ Pacemakers, deep brain stimulators (DBS), insulin pumps, endotracheal tubes, feeding tubes, indwelling catheters, colonoscopes, lumbar puncture needles, nasal swabs, and tongue depressors are all considered invasive. But these devices, and many others as well, differ in myriad ways – what they do, where they are located, what they are made of, or how long they last. Referring to devices as invasive may be a familiar part of medical and non-medical linguistic practice, but it is not clear exactly what it is about a device that makes it invasive or not. Despite pervasive use of the concept, a theoretical account of invasiveness is lacking.

The fact of an undertheorized concept of invasiveness is a little surprising given the outsized role the concept seems to play in bioethical discussions of medical devices. For example, invasiveness is offered as a reason to forego end of life treatments (e.g., intubation or feeding tubes (Spike 2012)), or to delay certain treatments until they are a ‘last resort’ (e.g., DBS (Stevens and Gilbert 2021), cardiac assist devices (Thiele et al. 2018)), or to limit biomedical research to animal studies or particular clinical populations (Gaillard 2017; Chiong et al. 2018). How the concept of invasiveness contributes to these ethical discussions may be a complicated story, but its rhetorical force is well-recognized (Gaillard 2017; Bluhm et al. 2021). Calling a device invasive matters to ethical discussions about devices.

In this paper, I will concern myself with understanding the concept of invasiveness of medical devices. The aim of gaining purchase on a concept of invasiveness is not, as I see it, to advance any particular ethical argument or to inform any particular scientific, regulatory, or bioethics debate. Instead, my focus is on the concept of invasiveness itself and how it can be understood in ways that are fruitful for debates across contexts. For the sake of simplicity, I will concern myself most directly with the use of the concept of invasiveness in and its implications for bioethics and the philosophy of medicine. That said, anyone involved in developing, funding, or prescribing medical devices, or for that matter, policy-making or communicating with the public about medical devices, would seem to benefit from a better understanding of what makes a medical device invasive.^4^

## Invasiveness of medical devices

As far as I can discern, *invasiveness* has no distinct or consensus meaning in medicine or biomedical science. Regulatory agencies do make reference to invasiveness. The European Union’s Regulation 2017/745 on medical devices, for instance, states that “‘invasive device’ means any device which, in whole or in part, penetrates inside the body, either through a body orifice or through the surface of the body” (EU 2017). The U.S. Food and Drug Administration (FDA) references the invasiveness of devices in their classification scheme, but does not define it (FDA 2006). There have been some attempts to build out from these regulatory uses of the concept. An example can be found in the field of neural engineering. Rajesh Rao, for instance, in his *Brain-Computer Interfacing: An Introduction*, defines invasive as involving “some form of surgery, wherein a part of the skull is removed, an electrode or implant placed in the brain, and the removed part of the skull then replaced” (Rao 2013, 18). Rao distinguishes between brain-computer interface (BCI) devices that are invasive (surgically implanted in the brain) and semi-invasive (surgically placed on the brain). Leuthardt et al. (2021) offer a different taxonomy of neural devices explicitly tied to gradations of surgical risk: invasive, minimally invasive (low risk of infection or tissue disruption), and minutely invasive (lower risk owing to introducing devices through non-surgical procedures like injection, ingestion, insufflation, or other non-surgical methods). These efforts are focused on categorizing devices, not giving an account of invasiveness.

### Introducibility

A reasonable place to start exploring a theoretical account of invasiveness is by identifying that to which invasiveness refers. A reasonable first candidate might be the way in which devices get into the body in the first place. Devices might be said to be introduced to the body in an invasive way. Surgery – cutting through the skin, scalp, bone or other tissues (Allison et al. 2010) - is the most obvious invasive procedure by which a device is put into the body. Invasive procedures are those “requiring the introduction of hands, instruments, or devices into the body via incisions or punctures of the skin or mucous membranes performed with the intent of changing the natural history of a human disease or condition for the better” (Ashton et al. 2009, 579). It seems reasonable, then, that a central feature of invasive devices might be that they are introduced to the body by some instrument(s), across some relevant bodily barrier(s), with a goal of improving bodily function(s) in some way. Or as Paul Ford and Abhishek Deshpande offer, an invasive device is “something that “transgresses” the body boundaries” (Ford and Deshpande 2013, 316).

As a first pass, introducibility does seem intimately connected to the concept of invasiveness. It is hard to think about an invasive device without at least wondering how the device gets into the body. That said, introducibility is not without its problems, as Rudnick (2011) points out. First, the introduction of something into the body is categorical – a device is either introduced into the body or it is not – and so introducibility “does not allow for a continuum, i.e., more or less invasiveness” (101). Yet invasiveness seems to be a concept that admits of degrees; some devices are more invasive and some less and some devices are non-invasive while others are minimally invasive. Second, introducibility “does not provide a sound rationale for distinguishing between mechanical objects and other objects, such as medications” (101). For instance, both an aspirin tablet and an endoscopy capsule device are introduced to the body in the same way (i.e., swallowing) but the latter seems invasive in a way that the former does not. While introducibility seems relevant to the invasiveness of devices, it is not clear that it can stand alone as a defining feature of invasiveness.

### Interiority

An alternative worth considering is whether what makes a device invasive is where a device is located – an invasive device might be said to do its work from the inside. Since how a device gets in and where it ends up often travel together, a simple thought experiment might be helpful to draw the contrast. Imagine that someone awoke to discover a cardiac pacemaker in their chest without a known history or physical evidence of that pacemaker ever having been implanted (e.g., no detectable scars). What would seem still to make this device invasive (besides having a person’s body accessed without consent^5^), is where the device is located - inside the body. The example highlights how facts about the way in which a device gets into the body might be largely beside the point. What might matter for invasiveness could just be where an invasive device is located and does its work – a device might be invasive in virtue of its interiority.

Of course, saying that what matters most to the concept of invasiveness is the line between what is internal and external to the body is one thing and being able to draw that line in a consistent and defensible way is quite another. For instance, no skin or other ectodermally-derived tissue is cut through or penetrated during the insertion of nasogastric or orogastric tubes, oxygen cannulas, endotracheal tubes, urinary catheters, thermometers, hearing aids, or IUDs. So, are none of these internal to the body? Or does the line between internal and external extend to orifices? Or does it need to extend further out still to devices impinging on sense organs (e.g., a laser shined in the eye during a laser assisted in situ keratomileusis (LASIX) procedure or a hearing aid placed in the ear)? Such examples may indicate that the search for a defensible line between what is inside and outside the body is quixotic, if not arbitrary.

### Foreignness

A third feature of medical devices that invasiveness might refer to is the way in which medical devices are foreign. A device might be considered invasive because it is not naturally part of the system into which it is introduced. This way of understanding invasiveness is in a way consonant with the etymology of the term ‘invasive’ (the medieval Latin *invāsīvus* term meant “invading” or “attacking”) which draws heavily on military metaphors (Oxford English Dictionary). A pacemaker is a foreign object put into the thorax. A DBS is a foreign object put into the brain. These devices are not *of* the thorax or *of* the brain. Invasive medical devices are often sources of infection or inflammation – the biological marker of foreignness. As such, it is worth considering whether what is central to the concept of invasiveness is that a medical device is a foreign object that changes the native system into which it is introduced, whether by bypassing or by taking control of native biological processes and structures.

While foreignness may be part of what is conveyed by the concept of invasiveness, it is hard to see it as the defining feature of what makes a device invasive. After all, medical devices of all kinds are foreign. People have foreign body reactions to most kinds of devices: electroencephalography (EEG) and electrocardiography (EKG) leads cause skin irritation, prosthetic limbs cause tissue erosion, and wearable patient monitoring devices cause local hair loss and skin changes. Yet, none of these devices are typically thought of as invasive. And even for a device that most might think is invasive, say a hypothetical future cardiac pacemaker constructed not out of metal and plastic but at least partly out of lab-grown human tissue, it is unclear how much of its foreignness (or reduction in such) relates to its invasiveness.

### Indelibility

A final candidate worth considering is the permanent effect that an invasive device has on the body. A device put into the body by cutting, burning, or penetrating human tissue leaves a mark. Davis and van Koningsbruggen (2013) and Glannon (2015) characterize invasive devices as those that cause tissue damage. Damage can result from how a device is put into the body (e.g., a scar develops where tissue was cut) but even the mere presence of a device in the body for an extended period of time can leave a mark, as evidenced by gliosis around devices in the brain (Vedam-Mai et al. 2018), neointimal lead encapsulation around pacemakers in the chest (Keiler et al. 2017), cellular overgrowth obstructing ventriculoperitoneal shunts (Blegvad et al. 2013)), or lipoatrophy around insulin pumps (Al Hayek et al. 2018). Even short-term use of devices can cause permanent change (e.g., vocal cord paralysis or granulomas from endotracheal tubes, skin burns from electroconvulsive therapy (ECT)). It is worth taking seriously that the central feature of an invasive device might be that it permanently changes the body: it leaves an indelible mark.

While indelibility can be important clinically or aesthetically or in other ways, it is not clear how central it is to the concept of invasiveness. Some devices leave relatively little in the way of perceptible marks on their users. Sometimes this is intentional (e.g., a surgeon works to hide incisions under hair lines or along skin folds) and sometimes this occurs by chance (e.g., the luck of a well-healed scar). And even when marks are perceptible, it is not clear how important they are: some device users find them insignificant, or forget about them over time, and some even celebrate and show them off. But perhaps the more relevant worry about indelibility is that it seems to be a feature of all medical interventions, not just invasive ones. Devices, medications, psychotherapy, for example, have their effects by changing the body in some way. Some of these changes are easily seen (e.g., scars) and others are more opaque (e.g., upregulated chemical receptors or new neural circuits). And while medical interventions are often described as temporary or reversible (“You can always stop the medication and go back to the way you were before.” Or: “The device can be explanted.”), this is not literally true. Every intervention leaves the body in a new state. As Heraclitus is credited with saying, “You can’t step twice into the same river” (Knowles 2009). ( The point here is simply that leaving a mark on or in the body seems neither specific to invasive devices nor consequential enough to be the defining feature of invasiveness.

It is worth taking stock now. Four plausible candidates for the concept of invasiveness have been surveyed (Table 1): (1) how a device gets placed (introducibility), (2) where a device is located (interiority), (3) whether a device belongs where it is placed (foreignness), and (4) whether a device causes damage or permanent change (indelibility). This survey is helpful for illuminating different ways in which people talk about invasive devices.^6^ But after this survey, it also seems clear that at the very least there is not a simple answer to the question of which of these features makes a device invasive. This raises a possibility worth considering: perhaps invasiveness is not a unitary concept at all.

**Table 1:** Descriptive features of invasive medical devices

	A device is medically invasive if it…	Descriptive question	Related terminology
**Introducibility**	…is introduced across some relevant barrier by some instrument.	How did device get there?	implanted, surgically placed, inserted
**Interiority**	…is located internally.	Where is device located?	Inside, internal
**Foreignness**	…is foreign to the body or tissue in which it works.	Does device belong there?	Foreign, mechanical
**Indelibility**	…permanently changes the natural order of biological structures, systems or processes.	What does device change there?	Irreversible, permanent, damage

Wittgenstein argues that concepts can have meaning even if it is not possible to provide necessary and sufficient conditions for their use (Wittgenstein 1958). Words and concepts, rather than having an essence, instead share a kind of family resemblance. Wittgenstein illustrates this view of language with the example of the idea of a game. Speakers can talk about games of various sorts – basketball, chess, charades, wrestling, fantasy role-playing, video – without there needing to be an essence that makes each of these a game. There is not one attribute that all games share, such as a ball, a net, or a referee. Rather, all things that are games share a kind of family resemblance. Their similarity is inexact and impossible to define for all instances, but there is enough overlap to support meaningful use of the concept.

What would applying a Wittgensteinian approach to language mean for the present discussion? At minimum, it might raise doubt about the wisdom of pursuing necessary and sufficient conditions for calling a medical device invasive. Features like introducibility, interiority, foreignness, and indelibility might be important for understanding invasiveness in general terms, but none of these might serve as the essence of invasiveness. Rather what makes the concept of invasiveness meaningful is that medical devices referred to as invasive share a family resemblance. This family resemblance is not definable in exact terms, but emerges from its use across medical contexts and devices. The features of introducibility, interiority, foreignness, and indelibility are highly relevant to understanding this family resemblance, but invasiveness is not reducible to them.

## Medical invasiveness as a thick normative concept

To this point, an assumption has been made that invasiveness is a technical or scientific concept of some kind. That is, the concept of invasiveness speaks to some descriptive question about a device: Where is it? How did it get there? Does it belong? What does it change? But people employ the concept of invasiveness in ways that don’t always seem to track these descriptive questions. Devices are described at times as “so invasive” or “too invasive” or “extremely invasive”. Employing the concept of invasiveness in these ways suggests that there may be something normative about calling a device invasive.

Concepts can be normative or non-normative. That is, while some concepts aim to describe the world as it is (i.e., descriptive concepts), other concepts are action-guiding and express values rather than mere facts about the world (i.e., normative concepts). Bernard Williams distinguishes between thick and thin normative concepts (Williams 1985). Thin normative concepts are action-guiding without needing to be world-guided; paradigmatic examples here include good, bad, ought, right, or wrong. Thin normative concepts provide evaluative direction (e.g., a reason for performing or not performing an action), but little else. Thick normative concepts, on the other hand, are both descriptive and evaluative. They provide evaluative direction tied to a rich descriptive content. Examples of thick normative concepts include courage, promise, brutality, and treachery (Williams 1985, 129). To say someone is cruel is more than just to offer a negative evaluation, it is to describe a characteristic way of behaving that intentionally inflicts suffering on another. While a number of ethical concepts have been proposed as thick, there has been increasing attention to thick normative concepts in other domains as well, such as aesthetics (e.g., garish), epistemology (e.g., gullible), and environmental ethics (e.g., resilience) (Väyrynen 2021).

This raises the possibility that perhaps the discussion to this point has involved an incorrect assumption about the very nature of the concept of invasiveness. What if invasiveness is a thick normative concept? That is, what if, when people call a device invasive, they are making, at least in part, an evaluative claim about the device? Just as calling an action “so cruel” (e.g., torturing animals) or calling someone “extremely courageous” (e.g., entering a burning building) is making a normative claim, perhaps calling a device invasive, with or without a qualifier, is also making a normative claim. If so, how might invasiveness be understood as a (thick) normative concept?

There is a weak way in which to interpret invasiveness as a thick normative concept. Invasiveness could simply signal or indicate that a medical device deserves some ethical attention. In this role, the concept of invasiveness could be a way of signaling that a medical device is of normative importance and deserves, at least prima facie, ethical scrutiny. Consider the following use case of biodegradable electronic devices (Shim et al. 2021). Sensors made of biodegradable materials are currently under development that may allow for temporary diagnostic monitoring, such as measuring intracranial pressure (e.g., traumatic brain injury), mechanical forces (e.g., tendon injury), or electrophysiological signals (e.g., epilepsy), or for targeted therapy, such as stimulating growth after injury (e.g., nerve damage) or delivering drugs (e.g., from heat-triggered degradable drug reservoirs). The absorption of implanted devices by the body after they have performed their function could obviate the need for surgical explantation and its associated risks. The development of biodegradable devices could be transformative.

Given the early stage of biodegradable electronics and its wide range of potential clinical applications, it is difficult to fully anticipate the range or type of normative implications. Maybe this technology would be intrusive or maybe not (e.g., require wearing protective equipment over the device). Maybe it would be disruptive of daily activities (e.g., avoiding physical activities that might damage or interfere with function of the device). Perhaps devices could be developed in ways that sidestep such normative concerns or invite others. The point is simply that it might be too soon to tell. In this case, then, calling this technology invasive might just be a way to signal both this uncertainty but also the importance of attending to potential moral implications, those that are readily apparent and those that would require analytic and empirical ethics exploration. Put more succinctly, the concept of invasiveness could be used to flag electronic devices made of biodegradable material as deserving of moral attention. This would be an important but admittedly modest role for the concept of invasiveness.

There is something about the weak interpretation of normative invasiveness that is unsatisfying. While the concept of invasiveness may draw ethical attention, it does not just do so in a general, abstract way. Rather, it draws attention to a range of specific ethical concerns. Namely, as will be argued next, invasiveness of devices implicates ethical concerns specifically related to dangerousness, intrusiveness, and disruptiveness. (Table 2).^7^

**Table 2:** Normative features of invasive devices

	A device is medically invasive if it…	Normative question	Related terminology
**Dangerousness**	…puts things of value at non-negligible risk of harm	How risky is the device?	Harmful, risky, unsafe
**Intrusiveness**	…transgresses some physical, mental, or social space	Does the device get in the way?	Violation, stigma
**Disruptiveness**	…disrupts an important aspect of the self	Is the person (not just their body) changed by using the device?	Alter (personality), change (identity), undermine (agency)

### Dangerousness

One way in which invasiveness functions as a normative concept is by conveying the dangerousness of medical devices. Medical devices risk causing harm. In most instances, the potential benefits of an invasive medical device outweigh risk of harm, but an invasive device is still dangerous to some extent. The connection between invasiveness and risk of bodily harm extends back to Greek medicine; rationalists preferred non-invasive therapies to invasive therapies owing to their lower risk (Matthen 1988). More recently, the risk of harm is taken to be central to the concept of medical invasiveness (Rudnick 2011; Glannon 2014, 2019; Leuthardt et al. 2021). The potential harms of invasive devices are described in physical terms - peri- or post-procedural hemorrhage, infection, tissue damage, or pain – but can also be psychological or social (Bluhm et al. 2021). The harms of invasive devices come in different degrees (more or less) or frequencies (rare or common) (Glannon 2014), and some have argued that the concept of invasiveness implicates a “least harm principle” such that the choice of an invasive device should be governed by a goal of less harm rather than more (Ford and Deshpande 2013).

### Intrusiveness: physical, mental, and social

Another way that invasiveness functions as a normative concept is by indicating that medical devices can be intrusive. Invasive devices intrude on physical, mental, or social spaces in undesirable ways. Ford and Deshpande (2013) argue that an invasive device “transgresses a person’s rights, interests, or personhood” and devices thereby “invade privacy, interfere with daily functioning, or invade one’s sense of self” (316). Some examples might be helpful here.

A medical device can be physically intrusive in obvious ways. A respirator in a person’s airway is uncomfortable and gets in the way of talking and eating. A dialysis machine and its tubes get in the way of freely moving about. But invasive devices can be physically intrusive in less obvious ways. The pulse generator for a pacemaker or implanted cardiac defibrillator (ICD) implanted just under the skin on the chest wall may not only interfere with some arm or shoulder movements but with the ability to comfortably wear certain clothing around others (e.g., bathing suits). Similarly, a responsive neurostimulator (RNS) under the scalp may interfere with the ability to comfortably wear hats. And even devices that are not perceptible by the user can still be physically intrusive. Some implanted devices (e.g., pacemakers, neurostimulators) interfere with the function of medical or non-medical devices (e.g., magnetic resonance imaging (MRI), airport screening machines). Not being able to access some kinds of medical care or having to go through extra security screening are kinds of physical intrusions (Goering et al. 2021; Klein and Rubel 2018).

Medical devices can also be mentally intrusive. This is perhaps most evident with devices that directly work on the brain. Increasingly there are biomedical devices that stimulate and change the function of the brain (DBS, transcranial magnetic stimulation (TMS)), read neural activity or its correlates out of the brain (EEG, functional magnetic resonance imaging (fMRI), BCI), or both (adaptive DBS (aDBS), RNS). These kinds of devices allow access to information about mental life that has not been possible before. When this access is unwanted, devices can be intrusive. Again, examples are helpful. A BCI device used to detect and infer subconscious processes, for instance inferring a bank personal identification number (PIN) (Bonaci et al. 2014), is mentally intrusive. Some have described this kind of intrusion in terms of violating mental integrity (Ienca and Adorno 2017) or cognitive freedom (Bublitz 2013). A less obvious example is found in the experience of some DBS users. DBS used to treat movement disorders can sometimes lead to changes in impulsivity (e.g., desire to gamble, hypersexuality) (Frank 2007). These induced desires, particularly if ego dystonic, can feel like an intrusion into a person’s mental life. But devices can be mentally intrusive even if they do not work directly on the brain. In her book *Lightning Flowers*, Katherine Standefer describes the mental intrusiveness of having an ICD that could fire or misfire at any moment. “I stood in coffee shops expecting to be shocked; I rode my bike expecting to be shocked; I warned my … students of my condition, in case something were to happen at the head of the room.” (Standefer 2020, 142).

Devices can be mentally intrusive not only by pushing their way into consciousness but by failing to recede from it. Work in the phenomenology of technology has highlighted ways in which devices can become embodied and transparent in their use (Ihde 2012; Dalibert 2016; Tbalvandany et al. 2019; McConville 2021). For instance, a person with an artificial hip may take a walk in the park, a person with a cochlear implant may engage in a captivating conversation, or a person with an insulin pump may partake in an enjoyable meal, all without giving thought in the moment to the devices that make these experiences possible. For them, the world is experienced transparently *through* these devices. Richard Heersmink (2013), drawing on the work of Merleau-Ponty and others, argues that medical devices, when functioning well, recede from awareness and become part of a person’s body schema. But some devices resist this transparency, either because their early stage of development impedes transparency (e.g., BCI control of neuroprosthetics (Heersmink 2013) or because devices interact with the human body in ways that inevitably intrude upon consciousness (e.g., an electric discharge from an ICD). Of course, the presence of a device also can push its way into consciousness by malfunctioning, wearing down, or becoming infected or obsolete, a type of experience that Hubert Dreyfus (1991) calls “breakdown”. When medical devices stop working as intended, they come out of transparency and become present to their users. This presence is an intrusion upon consciousness and reminds the user of the invasive device and its relation to their body.

Devices can also be socially intrusive. Non-medical devices, like mobile communication or recording devices, can be socially intrusive when used in public (e.g., in a theater or on public transit) (Kudina and Verbeek 2019). Medical devices, too, can be socially intrusive. A person with a pacemaker pulled out of line at airport security experiences not just the physical intrusion of a device - they cannot separate themselves from the device and put it in their carryon luggage, for instance – but a kind of social intrusion as well. The very fact of having a medical device attracts unwanted attention and can be a potential source of stigma (Aas 2016; Goering et al. 2021). Medical devices can be socially intrusive in more intimate ways. Consider how a speech-enabled glucose monitor might report a blood sugar value to all within earshot (and hence reveal a user’s recent dietary discretions)^8^ or how an assistive communication device used by someone with amyotrophic lateral sclerosis (ALS) might broadcast a user’s speech act (e.g., a desire for hygiene care) to intimates and strangers alike (Klein et al. 2022). Or consider the case described by Klein et al. (2016) of a young woman enrolled in a DBS trial for treatment of depression who argues often with her father. During one argument, he suggests that the source of their conflict might be that she needs her DBS settings “turned up”, while she protests that the source of their conflict lies elsewhere. While their arguments predate getting the DBS device, what is notable is that the DBS is now part of – in the middle of – their relationship. It has become socially intrusive.

### Disruptiveness

Another way in which invasiveness functions as a normative concept is by indicating how medical devices can be disruptive, not just of the body (i.e., altering bodily structure and function – the indelibility point noted previously), but of the self. Medical devices can lead to unwanted changes in how individuals understand themselves or are understood by others. For example, DBS has been reported to cause certain changes in personality and subsequent marital conflict and occupational effects (Schupbach et al. 2006). Some have described the disruptions experienced by DBS users in terms of changes in narrative identity (Schechtman 2010; Baylis 2013) or agency (Goering et al. 2017). Others have described how device users experience disruptions in their sense of vulnerability (Goering et al. 2021; Ford 2009; Humphreys 2016) or day to day activities, what Bluhm et al. (2021) call “lifestyle” invasiveness. And yet others describe how medical devices of various kinds can put stress on or undermine the relationships between device users and their loved ones (Campelia et al. 2019; Thomson et al. 2020).

The implications of taking seriously the idea that invasiveness is a normative concept may be wide-ranging. Not only may it require that greater care be taken in using the concept going forward, it also may invite a reexamination of language used in longstanding debates in bioethics and the philosophy of medicine. For instance, feeding tubes have been called invasive devices in debates about ethical treatment of people with advanced dementia (Post 2001; Sampson 2010), Within these debates, referring to devices as invasive has been taken to be part of the preparatory work of laying out relevant facts. These facts are taken as the starting point for ethical argument. But if the argument advanced here is correct, then calling a feeding tube invasive is already making a normative claim of some kind. Whether that normative claim is related to the dangerousness of the device, the disruption it causes, or its intrusiveness, or something else is a further question. The point here is just that if invasiveness is a thick normative concept then how it is gets used in discourse about medical devices matters and may need greater attention.

## Medical invasiveness: future directions

Before concluding, it is worth thinking about whether there are resources that can be drawn upon for working out a richer understanding of invasiveness as a thick normative concept. While work on thick normative concepts in ethics may be useful (Väyrynen 2013) as may work related to the concepts of dangerousness, intrusiveness, and disruptiveness (Bury 1982; Dworkin 1977), there are other, more proximal resources worth noting.

One such resource is discussion about neurotechnology and the extension of mind. Joel Anderson (2008) and Tom Buller (2013), for instance, argue over whether adopting a view of the mind as extended beyond the body has moral implications for evaluating neurotechnology, like neuroprosthetics. Anderson argues that since the “skin-and-skull boundary” lacks metaphysical and ethical significance, an “invasiveness criterion” (i.e., that physical invasions matter morally) should be rejected. Buller counters that a functional understanding of the boundary between person and body, rather than a biological understanding, warrants retaining the invasiveness criterion. The details of their respective arguments matter less for our purposes than the fact that in the midst of this debate they grapple with the descriptive and normative dimensions of invasiveness. For instance, in advancing his argument Buller refers to an invasive intervention as one that “causes the person pain and suffering, or renders her more vulnerable to further injury, or restricts her freedom or autonomy” (594). This is the kind of definition that may be a promising starting point for developing a robust account of invasiveness as a thick normative concept.

Another resource can be found in the philosophy of medicine. The same normative concepts – danger, intrusion, disruption – are also a part of everyday medical practice. The clinical encounter – the various ways in which patients and clinicians interact – is, in many ways, invasive. Touching during a physical exam and the intimacy of questions asked about medical, social, and family history are intrusive. Taking a new medication or undergoing surgery is dangerous. Finding out a new diagnosis and embarking on therapy is disruptive of daily life, relationships, and often one’s self-conception. It is because the practice of medicine is inextricably bound up with these and other normative concepts that the practice of medicine is fundamentally a normative practice (MacIntyre 1981; Pellegrino 1995; Miller and Brody 2001). As such, the philosophy of medicine itself may be an additional resource for working out the normative content of invasiveness.

Finally, asking medical device users themselves about their experience can illuminate the concept of invasiveness. For instance, Bluhm et al. (2021) conducted semi-structured interviews with 48 individuals (including 16 patients) who had experience with medical devices treating major depressive disorder (ECT, TMS, DBS) about their views of invasiveness. The study found that interviewees recognize three different kinds of invasiveness of these devices: physical, emotional, and lifestyle. McCall et al. (2020) conducted semi-structured interviews with 17 patients and clinicians involved in a first-in-human trial of a novel device for performing ablative brain surgery (magnetic resonance-guided focused ultrasound) about their views of the device, including its invasiveness. The study found differences in how clinicians and patients understood invasiveness: all clinicians described the device as invasive, but only half of patients did so. One clinician described the device as invasive in virtue of it causing “irreversible damage to the brain”, whereas one patient explained that they considered the device non-invasive because it was “not real surgery”. These studies demonstrate the potential value of talking directly to users of devices about the meaning of invasiveness.^9^

## Conclusion

It has been argued here that the concept of invasiveness is frequently employed in bioethics and the philosophy of medicine, but without an adequate theoretical basis. There are four descriptive features commonly associated with invasive medical devices - introducibility, interiority, foreignness, and indelibility. None of these provide a definition of invasiveness, and the concept invasiveness is best thought of as a kind of family resemblance shared across medical devices. This concept of invasiveness is thick normative concept, one that conveys the potential dangerousness, intrusiveness, or disruptiveness of a medical device.

## Footnotes

1 This is to distinguish it, for present purposes, from a more general concept of invasiveness. Invasiveness is a concept with meaning across a broad range of contexts, from ecology (e.g., invasive species), to information technology (e.g., privacy invasion), to criminal justice (e.g., invasive searches) to others. Medical invasiveness is meant to bracket for the present discussion use of the term invasiveness and its cognates in reference to medical devices. This bracketing does not presume that how the concept of invasiveness is used in other contexts is not relevant to discussion of invasiveness of medical devices. But it does presume that it makes sense, and that there is some value, to a narrow discussion about invasiveness of medical devices. Since the subsequent discussion will be confined to the medical context, I will sometimes refer to ‘invasiveness’ rather than using the longer term ‘medical invasiveness’.

2 For simplicity, I will use the adjective ‘invasive ‘and the noun ‘invasiveness’ interchangeably.

3 Here I adopt the World Health Organization (WHO) definition of a medical device as “an article, instrument, apparatus or machine that is used in the prevention, diagnosis or treatment of illness or disease, or for detecting, measuring, restoring, correcting or modifying the structure or function of the body for some health purpose. Typically, the purpose of a medical device is not achieved by pharmacological, immunological or metabolic means” (WHO 2022).

4 I regretfully acknowledge that my use of the concept of invasiveness at times has lacked precision.

5 In this respect, the example is reminiscent of Judith Jarvis Thomson’s (Thomson 1971) thought experiment in which a pregnant woman awakens to find herself plugged into the circulatory system of a famous violinist with a fatal kidney ailment.

6 This exploration may also be helpful for understanding use of the concept in non-device medical contexts, for instance in immunology, oncology, psychiatry, and transplant medicine, though this is beyond the scope of the current paper.

7 This list is not meant to be exhaustive of the normative implications of invasive medical devices.

8 I would like to thank Sara Goering for this example.

9 Felicitas Kraemer (Kraemer 2013) argues that attending to phenomenological experiences with invasive devices may be a way to help move debates in bioethics past ontological impasses, for instance, the meaning of passive euthanasia in deactivation of left ventricular assist devices.
